# Development of an electrochemiluminescence-based bridging assay to detect antibodies against a PTH inverse agonist in human plasma

**DOI:** 10.1080/17576180.2026.2709280

**Published:** 2026-08-10

**Authors:** Asvelt J. Nduwumwami, Erik J. Wagner, Amy Q. Wang, Yuhong Fang, Dingyin Tao, Xin Xu

**Affiliations:** National Center for Advancing Translational Sciences, National Institutes of Health, Rockville, MD, USA

**Keywords:** Anti-drug antibody (ADA) assay, Jansen’s Metaphyseal Chondrodysplasia (JMC), parathyroid hormone inverse agonist (PTH-IA), liquid chromatography-mass spectrometry (LC-MS), screening assay cut point, confirmatory assay cut point

## Abstract

**Background:**

Therapeutic peptide-induced anti-drug antibodies (ADA) can have various effects, including adverse events and drug-neutralizing activity which can lead to loss of efficacy. This has led regulatory agencies to require nonclinical and clinical testing of biotherapeutics to assess immunogenicity risk.

**Research design and methods:**

An electrochemiluminescence bridging assay was developed to detect antibodies against parathyroid hormone receptor inverse agonist (PTH-IA), a novel synthetic peptide that is being developed to treat Jansen’s Metaphyseal Chondrodysplasia. Biotinylated PTH-IA and ruthenylated PTH-IA reagents were synthesized via amine-coupling chemistry, and rat polyclonal anti-PTH-IA positive control antibody was generated by immunizing rats with PTH-IA conjugated to keyhole limpet hemocyanin. The reagents were utilized to determine cut points for the screening and confirmatory assays and assay sensitivity in drug-naïve human plasma.

**Results:**

Sensitivity of the screening assay was determined to be 3.61 ng/mL at cut point. The cut point for the confirmatory assay was determined to be 9.34% inhibition, and the cut point factor used to establish the floating plate cut point from the negative control signal was 1.055. The assay was found to be selective and reproducible.

**Conclusion:**

The assay’s performance characteristics demonstrated its suitability for detecting anti-PTH-IA antibodies in human plasma.

## PLAIN LANGUAGE SUMMARY

Synthetic versions of the body’s hormones are used as medicines to treat genetic diseases. In some patients, these medicines can activate the immune system and cause the body to produce antibodies. These antibodies can, in turn, affect the effectiveness of the medicines or cause adverse effects. Therefore, reliable laboratory tests that can accurately detect these treatment-induced antibodies are required to provide insights into the efficacy and safety of these types of medicines.

In this study, we developed a plasma-based sensitive test that detects antibodies against a novel synthetic hormone known as PTH-IA. PTH-IA is being developed to treat an ultra-rare genetic disease of the bone and mineral ion homeostasis called Jansen’s metaphyseal chondrodysplasia. We used a reference antibody to evaluate the reliability of the test and assessed the impact of the body’s natural substances that have some similarity to PTH-IA on performance of the test in human plasma. We found that the test can reliably detect antibodies against PTH-IA at levels that are as low as 3.61 ng/mL. The test was also specific and showed that two structurally similar hormones, parathyroid hormone and parathyroid hormone-related peptide, did not interfere with its performance.

The ability of this test to distinguish negative samples from those that are positive for anti-PTH-IA antibodies implies that it can help clinicians identify patients that are not responding to PTH-IA treatment because their bodies are producing anti-PTH-IA antibodies. It can also assist doctors in designing mitigation strategies in case of adverse events caused by anti-PTH-IA antibodies.

## Graphical Abstract

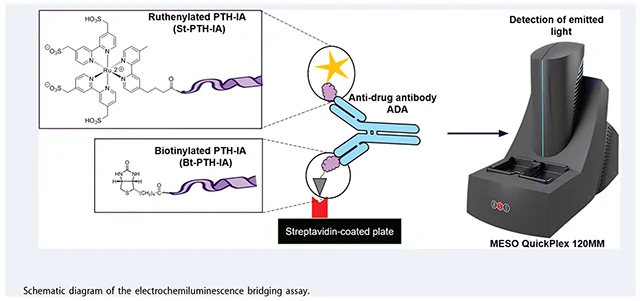

## 1. Introduction

Therapeutic peptides have unique physico-chemical features, including a large surface area and a flexible backbone that enable them to modulate biological processes mediated by ligand-receptor or protein–protein interactions [1,2]. Their application spans a wide range of therapeutic indications, including oncology, cardiovascular and metabolic diseases, and they are good candidates for extracellular targets such as G protein-coupled receptor (GPCR) [3–5]. Parathyroid hormone receptor-1 (PTHR1) is a B family GPCR that interacts with its endogenous peptide ligands, parathyroid hormone (PTH) and PTH-related peptide (PTHrP), to mediate pleiotropic effects in the body. It regulates normal bone growth by slowing chondrocyte differentiation [6], maintains homeostasis between the resorptive and anabolic activities of osteoclasts and osteoblasts [7], and keeps blood calcium and phosphate ion levels in normal ranges [8].

Constitutive signaling of PTHR1 has been implicated as the cause of Jansen’s Metaphyseal Chondrodysplasia (JMC), an ultra-rare disease characterized by skeletal abnormalities and progressive impairment of renal function [9]. Analysis of genomic DNA of JMC patients identified five activating PTHR1 mutations. These mutations, H223R, T410P, T410R, I458K, I458R, have been shown to slow chondrocyte maturation leading to abnormal bone growth, and to induce ligand-independent hypercalcemia and elevated phosphate excretion, both of which are associated with increased bone resorption [10,11]. Current medical interventions include low-calcium and low-salt diet, oral phosphate supplementation, recurrent surgeries to realign and reshape bones and joints, or nephrolithotomy for renal calcifications [10]. However, these interventions do not specifically target the abnormal signaling of PTHR1 mutants.

Parathyroid hormone inverse agonist (PTH-IA) is a 30-amino acid synthetic peptide derived from PTHrP through truncation, insertion of non-PTHrP native amino acids including substitution of L-Gly 12 with D-Trp. It exhibits potent inverse agonism to all five JMC- causing PTHR1 mutants and has been shown to reverse PTHR1 constitutive activity in cellular functional assays [10] and to partially rescue skeletal deformities in murine models of JMC [12]. Therapeutic peptides such as PTH-IA are, however, prone to inducing generation of antidrug antibodies [13]. Additionally, the modifications aimed at increasing the therapeutic performance of PTH-IA such as truncation and introduction of unnatural amino acids have been reported to enhance the immunogenicity risk [14]. Effects of unwanted treatment-induced anti-drug antibodies have been reported [14–38]. They include alteration of pharmacokinetic profile due to ADA-drug complex formation that can result in increased drug clearance [23], neutralization of drug activity in cases where ADA prevents drug–target interaction [38], or adverse events due to ADA cross-reaction with endogenous targets [17,18]. These effects can complicate interpretation of clinical trial results and require biologic drug monitoring to include assessment of immunogenicity. Thus, a bioanalytical assay for the assessment of potential antibodies against PTH-IA is required to support efficacy and safety evaluations in clinical trials.

Various immunoassay formats for the detection of anti-drug antibodies exist and their advantages and disadvantages have been discussed in the literature [24,32]. We chose electrochemiluminescence-bridging format due to its high sensitivity, stability of ruthenylated reagent, and superior dynamic range over other commonly used immunoassays [24,26]. In this manuscript, we describe the development of an electrochemiluminescence-based immunogenicity assay to detect anti–PTH-IA antibodies in human plasma.

## 2. Materials and Methods

### 2.1. Reagents and materials

PTH-IA was produced by Bachem (Torrance, CA). The rat polyclonal antibody against PTH-IA (pAb) used to develop the ADA assay was generated by Precision Antibody, Inc (Columbia, MD) by immunizing rats with PTH-IA conjugated to Keyhole Limpet Hemocyanin (KLH), pooling sera from high titer individuals followed by affinity purification with agarose-PTH-IA and elution by glycine. Human PTHrP and PTH (1–84) peptides were purchased from GoldBio (St. Louis, MO). Individual human K_2_EDTA plasma samples from healthy male and female drug naïve donors were purchased from BioIVT (Westbury, NY). 20X Phosphate Buffered Saline (PBS) with 1% Tween-20 (20X PBST) was from Teknova. EZ-Link Sulfo-NHS-Biotin, plate sealers, superblock in PBS, and acetonitrile were purchased from Thermo Fisher Scientific (Waltham, MA). Formic acid and dimethylsulfoxide (DMSO) were obtained from Sigma (Rockville, MD). MSD Read Buffer T (4X), MSD GOLD SULFOTAG NHS-Ester and MSD GOLD 96-well Streptavidin SECTOR plates were purchased from Meso Scale Discovery (Gaithersburg, MD).

### 2.2. Generation and characterization of biotinylated-PTH-IA and ruthenylated-PTH-IA reagents

PTH-IA was biotinylated by incubating it with 15X molar excess of EZ-Link Sulfo-NHS-Biotin in 30% DMSO in 1X PBS for overnight at 4°C. After completion of the conjugation reaction, a precipitate formed. The reaction mixture was spun at 1200 × g at 4°C for 10 min, the supernatant was transferred to a clean tube and the remaining pellet was washed with 30% DMSO in 1X PBS three times. The pellet was reconstituted in a solution containing 50% acetonitrile and 1% formic acid in MilliQ water. For the ruthenylation reaction, PTH-IA was incubated with 10X molar excess of MSD GOLD SULFO-TAG NHS Ester in 30% DMSO in 1X PBS for overnight at 4°C while protected from light. A homogenous mixture was obtained after the conjugation reaction. Aliquots of unconjugated PTH-IA, sulfo-tag labeled PTH-IA mixture, dissolved pellet and supernatant from the biotinylation reaction were subjected to HPLC-TOF MS analysis with a protein column. Liquid chromatography (LC) was performed on an Agilent 1290 Infinity II LC system (Agilent Technologies, Wilmington, DE) equipped with a diode array detector (DAD), binary pump, multicolumn thermostat, and autosampler. The mobile phases used for the separation were MS-grade water with 0.1% formic acid (solvent A) and MS-grade acetonitrile with 0.1% formic acid (solvent B). Elution of analytes from the analytical column (HALO BioClass Protein Diphenyl, 1000 Å, 2.7 μm, 2.1 × 100 mm) was performed using a gradient starting at 5% B at a flow rate of 0.4 mL/min. The mobile phase was then 5–15% B for 1 min, 15–45% B for 3.5 min, 45–90% B for 0.5 min and maintained at 90% B for 1.5 min, followed by 5% B for 0.5 min and re-equilibration of the column with 5% B for 1.4 min. Chromatographic separations were performed at a column temperature of 60°C with a total run time of 8 min. Two microliters of each sample were injected onto the column for LC-MS analysis. Mass spectrometry (MS) experiments were conducted on an Agilent 6230 TOF system (Agilent Technologies, Wilmington, DE), equipped with a DUAL AJS ESI source operating in positive ion mode. MS spectra were acquired from m/z 300 to 3000 at a scan rate of 1 spectrum per second with Profile format. The electrospray ionization (ESI) source parameters were as follows: gas temperature: 325°C, gas flow: 8 L/min, nebulizer: 35 psi, sheath gas temperature: 350°C, Vcap: 3500 V, nozzle: 1000 V, fragmentor: 175 V. HPLC-TOF data analysis was performed by Agilent MassHunter Qualitative Analysis (B.07.00).

### 2.3. ADA bridging assay procedure

A checkerboard assay determined the optimal final concentrations of biotinylated PTH-IA (Bt-PTH-IA) and ruthenylated-PTH-IA (St-PTH-IA) to be 0.1 μg/mL in the ADA assay in the presence of 5 μg/mL of anti-PTH-IA pAb spiked in 10% of a pool of human plasma. To determine the minimum required dilution (MRD), we evaluated a series of standard curves of anti-PTH-IA pAb spiked in undiluted human plasma or human plasma diluted in PBST at 1:3, 1:6, 1:9, 1:12, and 1:15. The MRD of 1:3 provided the best sensitivity and was chosen to be used in assessing ADA assay performance characteristics. The rest of the assay procedure was as follows: positive and negative control (NC) samples and drug-naïve human K_2_EDTA plasma from healthy male and female donors were diluted in 1X PBS superblock, pH 7.4, at the selected MRD of 1:3. 60 μL of each diluted sample or control was combined with 60 μL of a master mix prepared by combining 30 μL of 0.2 μg/mL Bt-PTH-IA and 30 μL of 0.2 μg/mL St-PTH-IA and incubated at room temperature on a plate shaker set at 500 RPM for 1 h. Concurrently, MSD Gold streptavidin-coated 96 well plate was blocked by adding 150 μL of PBS superblock to each well and incubated at room temperature on a plate shaker at 500 RPM for 1 hour. After removing PBS superblock from the MSD Gold streptavidin-coated 96 well plates, an aliquot of 100 μL of the incubated samples and controls was transferred to each well of the blocked streptavidin plate and placed on a plate shaker at 500 RPM for 1 hour. The plate was washed three times with 1X PBST and 150 μL of 2X MSD Read Buffer T was added to each well to develop the signal. The plate was read using an MSD SQ-120 plate reader (Meso Scale Discovery, Gaithersburg, MD).

### 2.4. Determination of cut points

A panel of 50 drug-naïve human K_2_EDTA plasma samples from healthy male and female donors was used to determine cut points. The samples were screened in duplicate in two independent runs. Signal from individual sample was normalized by dividing the mean signal of the replicates by the mean NC signal on the same plate, ROUT analysis (Graphpad Prism) was performed to identify outliers, and normal distribution of results was assessed by the Shapiro–Wilk test after removal of outliers [16,30]. A plate specific floating cut point (SCP) for the screening assay was determined by the parametric approach: SCP = mean +1.645 × SD [19,21], where mean is the average of sample signals and SD is the standard deviation. The screening cut point factor (CPF) was determined by signal to noise (S/N) ratio and a SCP for each subsequent plate was determined by multiplying the mean NC by CPF.

The confirmatory assay was performed using the same panel of plasma samples spiked with 4 μg/mL of unlabeled PTH-IA while maintaining 1:3 final dilution of plasma in 1X PBST. After incubation of sample: unlabeled PTH-IA mix at room temperature on a plate shaker at 500 RPM for 1 hour, an aliquot of 60 μL sample/PTH-IA mix was transferred to a 96 well polypropylene plate containing 60 μL/well of master mix, and the rest of the assay proceeded in the same manner as the screening assay. Percent signal inhibition for each sample was determined using the following equation as described in [19]:

%signal inhibition=100×[1-(PTH−IA spiked sample signal/unspiked sample signal)]

The confirmatory cut point (CCP) was calculated using the parametric approach at 1% false positive rate: CCP = mean +2.33 × SD, where mean is the overall average of the percent signal inhibition within the panel and SD is the standard deviation of the percent signal inhibition estimates [19].

### 2.5. Assessments of drug tolerance and precision of the screening and confirmatory assays

Drug tolerance was assessed in two independent experiments according to the following procedure. Aliquots of a pool of 50 drug-naïve human K_2_EDTA plasma samples from healthy donors spiked with 10 ng/mL of anti-PTH-IA pAb at a minimum dilution of 1:3 were preincubated with serial dilutions of unlabeled PTH-IA at room temperature, shaking at 500 RPM for 1 hour. A master mix containing a mixture of St-PTH-IA and Bt-PTH-IA was then combined with an equal volume of the sample/ PTH-IA blend in 96-well plate and incubated at room temperature on a plate shaker at 500 RPM for 1 hour. An aliquot of 50 μL/well of each sample (in triplicate) was transferred to a streptavidin-coated plate followed by incubation at room temperature with shaking at 500 RPM for 1 hour. After incubation, plates were read on the MSD SQ-120 instrument as before. Mean ECL values were plotted on a 5-PL non-linear curve and drug tolerance was determined by interpolation of the floating plate cut point. Screening assay precision was evaluated using quality control samples in six replicates. The NC was prepared by pooling drug-naïve normal human K_2_EDTA plasma samples. The low (LPC), medium (MPC), and high (HPC) positive controls were 12.5, 100, and 1250 ng/mL of anti-PTH-IA rat polyclonal antibody spiked in pooled human plasma. The NC and the three levels of PC concentrations were mixed with an equal volume of the master mix of ruthenylated and biotinylated PTH-IA reagents. For the reproducibility of the confirmatory assay assessment, the same quality controls as in the screening assay were preincubated with 4 μg/mL of unlabeled PTH-IA for 1 hour at room temperature with shaking at 500 RPM before they were combined with the master mix of reagents. The rest of the assay proceeded as in section 2.3.

## 3. Results

### 3.1. Biotin/sulfo-tag conjugation of PTH-IA using amine coupling chemistry to generate assay reagents

Detection of antibodies specific to PTH-IA was carried out on the MSD platform. The setup for this ADA bridging assay involved incubating samples with Bt-PTH-IA and St-PTH-IA to allow formation of anti-PTH-IA antibody/Bt-PTH-IA/St-PTH-IA complex. The ternary complex was then captured onto a streptavidin-coated plate and bound complex was quantitated by measuring electrochemiluminescence signal (ECL). To develop the assay, biotin- and sulfo-tag-conjugated PTH-IA critical reagents were required. Both EZ-Link Sulfo-NHS-Biotin and MSD GOLD SULFO-TAG-NHS Ester possess N-Hydroxysuccinimide (NHS) ester groups that allowed coupling these tags to PTH-IA through its internal lysine residue and amino terminus. One challenge was that PTH-IA is soluble in acidic conditions while both conjugation reactions occur within a mildly basic pH range 7–9. To maximize the labeling efficiency, we examined various combinations of buffer, pH, reaction incubation time, temperature, and conjugation ratio. We achieved best results by dissolving PTH-IA in 1X PBS solution at pH 8 containing 30% DMSO followed by incubation with either EZ-Link Sulfo-NHS-Biotin or MSD GOLD SULFO-TAG-NHS Ester at 15:1 (EZ-Link Sulfo-NHS-Biotin to PTH-IA) or 10:1 (MSD GOLD SULFO-TAG-NHS Ester to PTH-IA) molar ratio at 4°C for overnight. Under these optimized conditions, a precipitate formed in the EZ-Link Sulfo-NHS-Biotin/PTH-IA conjugation reaction. The pellet was separated from the supernatant by centrifugation, washed with 30% DMSO in 1X PBS at pH 8 and reconstituted in a solution containing 1% formic acid and 50% acetonitrile in H_2_O. The supernatant and the reconstituted samples were analyzed by LC-MS to characterize free and conjugated PTH-IA. Unlike the biotinylation reaction, no precipitate formed in the MSD GOLD SULFO-TAG-NHS Ester/PTH-IA conjugation reaction due to multiple charges added to the ruthenylated PTH-IA product.

### 3.2. Characterization of Bt-PTH-IA and St-PTH-IA by reverse phase liquid chromatography coupled to TOF mass spectrometry (LC-MS)

**Figure 1.**
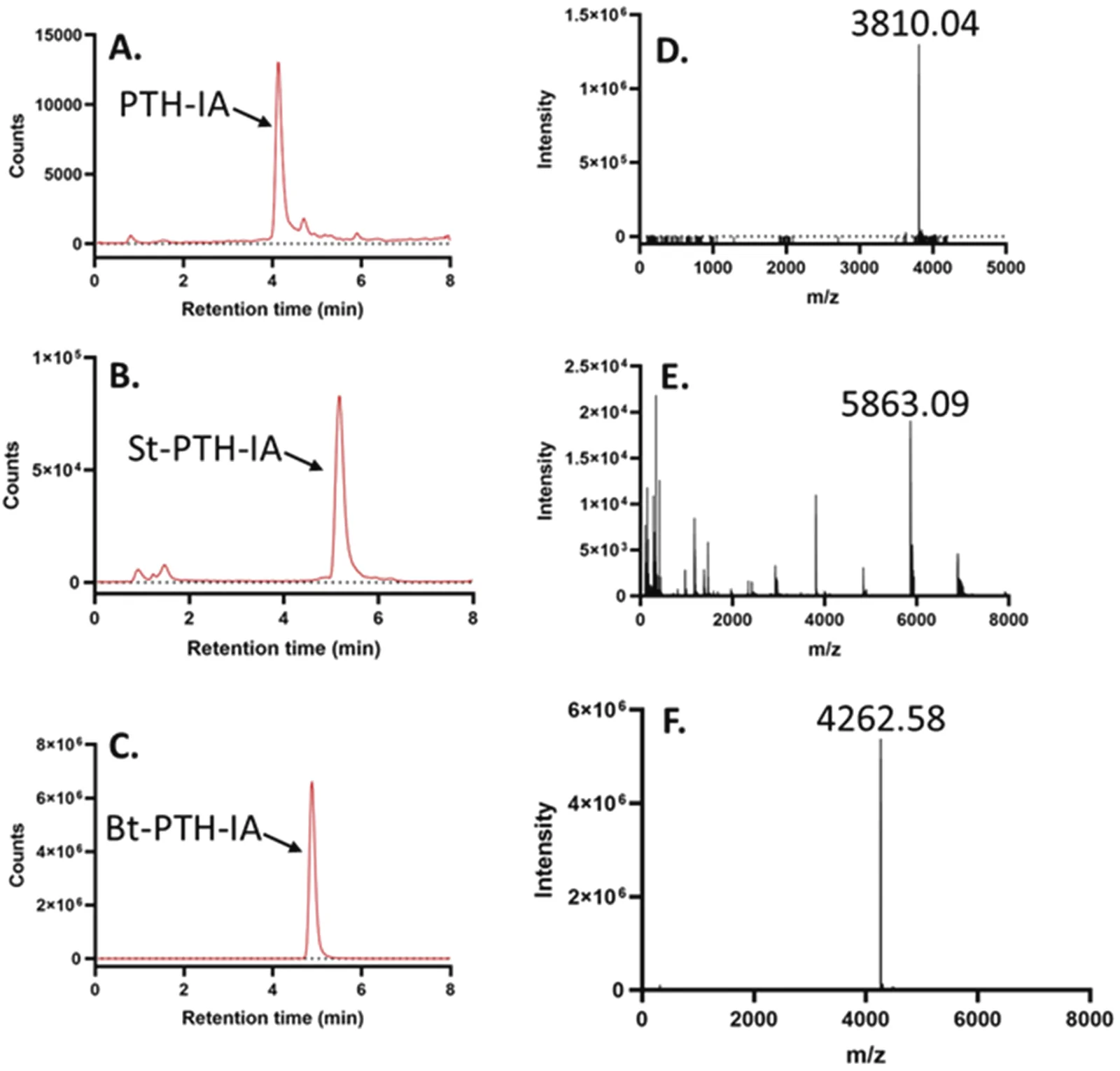
LC-MS analysis results of intact and conjugated PTH-IA. Extracted ion chromatograms of free PTH-IA (A), sulfo-tag labeled PTH-IA (St-PTH-IA) (B), and biotinylated PTH-IA (Bt-PTH-IA) (C). Deconvoluted mass spectra of free PTH-IA (D), St-PTH-IA (E), and Bt-PTH-IA (F).

LC-MS analysis performed on PTH-IA before conjugation identified a chromatographic peak at retention time (RT) of 4.17 min (Figure 1(A)) with a deconvoluted mass spectrum showing a molecular weight (MW) of 3810 (Figure 1(D)) that matched the expected MW of free PTH-IA. This confirmed the identity of the starting material as PTH-IA, and both RT and MW were used to assess whether the conjugation reactions proceeded to completion. Two molar excess of MSD GOLD SULFO-TAG-NHS Ester (challenge ratio 2:1) produced a heterogenous mixture of unmodified PTH-IA, singly- and doubly- labeled PTH-IA products. The single and double labeling were consistent with the amine coupling chemistry as PTH-IA has two conjugation sites: one internal lysine residue and an amino terminus. Increasing the challenge ratio to 10:1 resulted in completion of the conjugation reaction with incorporation of 2 sulfo-tag labels per PTH-IA molecule (Figure 1(B,E)). Separation of free sulfo-tag from conjugated PTH-IA was not necessary, as free sulfo-tag is removed during the final wash step.

Biotinylation reaction at a 15:1 challenge ratio resulted in a 99.8% doubly-biotinylated PTH-IA (Figure 1(C,F)). The 0.2% unreacted PTH-IA was deemed too low to interfere with assay performance (see section 3.7 on drug tolerance). No EZ-Link Sulfo-NHS-Biotin peak was observed in the total ion chromatogram of Bt-PTH-IA pellet sample, indicating that pellet washes coupled with centrifugation removed residual biotin. The doubly labeled reagents, as indicated by deconvoluted mass spectra peaks at MW of 5863.09 for St-PTH-IA (Figure 1 (E)) and 4262.58 for Bt-PTH-IA (Figure 1(F)), were used at a final concentration of 0.1 μg/mL each to develop the ADA assay. Several parameters we evaluated included sensitivity and cut points for the screening and confirmatory assays.

### 3.3. Screening assay cut point

**Figure 2.**
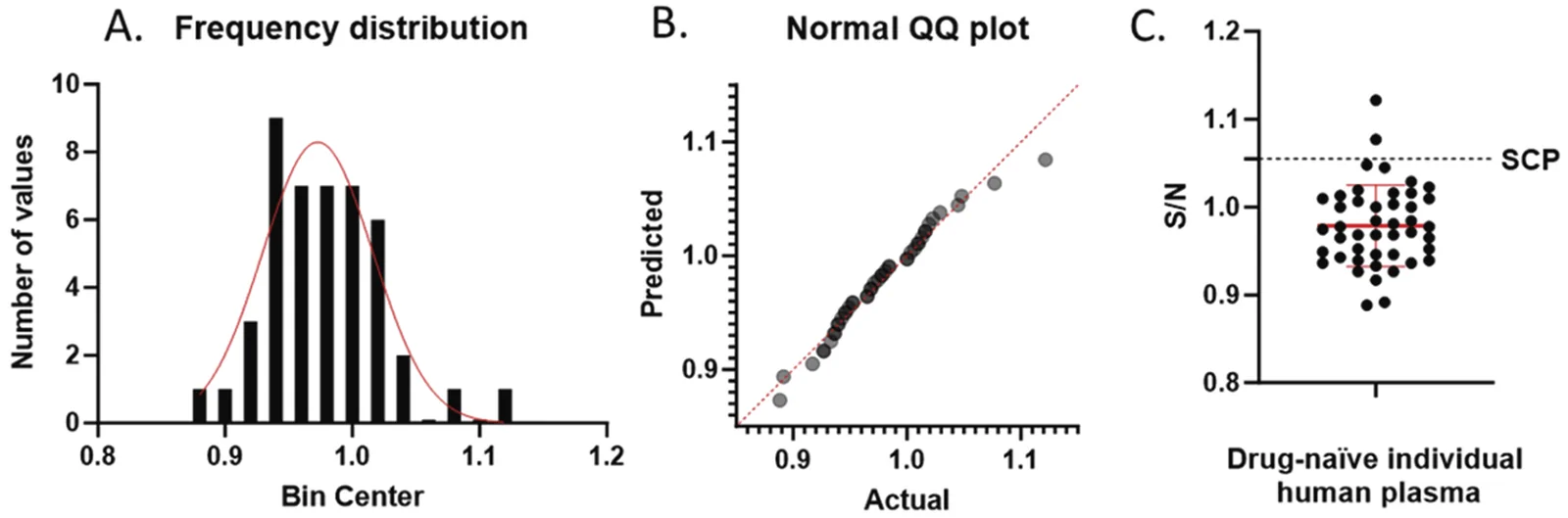
Determination of floating screening cut point: 50 drug-naïve human plasma samples were assayed in duplicate wells in two independent runs of the screening assay and mean of results normalized to the mean negative control to determine screening assay cut point. Distribution of drug-naïve human plasma sample results without the outliers (A), Quantile–Quantile (QQ) plot of actual distribution versus predicted normal distribution (B), and scatter plot of normalized results, mean ± SD in red (C). Dashed line indicates screening assay cut point (SCP).

Human plasma samples from 50 healthy drug-naïve individuals were utilized to assess inter-subject and assay variability at the selected MRD of 1:3 and to determine the screening cut point (SCP) of the assay. An assay SCP is defined as the level of response at or above which samples are classified as potentially positive for ADAs and below which they are considered negative [16,19]. Two independent runs were conducted in duplicate wells for each plasma sample, and the ECL values were averaged and subsequently divided by the mean of the NC matrix pool. An initial Shapiro–Wilk normality test carried out on drug-naïve sample results indicated a non-normal distribution even after transforming the results to a logarithmic scale. This suggested presence of outliers. The ROUT analysis performed on normalized results identified five outliers which were removed to avoid inflating variability and screening cut point. The remaining 45 normalized values exhibited a normal distribution without data transformation (Figure 2(A)) as confirmed by the Shapiro–Wilk normality test (*p* = 0.3014) and the quantile–quantile (QQ) plot (Figure 2(B)). The cut point was set at a 5% false positive rate and determined to be 1.05 in S/N (Figure 2(C)), which corresponded to 109.7 ECLU. The cut point factor (CPF) of 1.055 was determined by dividing 109.7 ECLU by the plate’s NC at 104 ECLU and was used in the determination of screening cut point for subsequent runs.

### 3.4. Sensitivity of the screening assay

**Figure 3.**
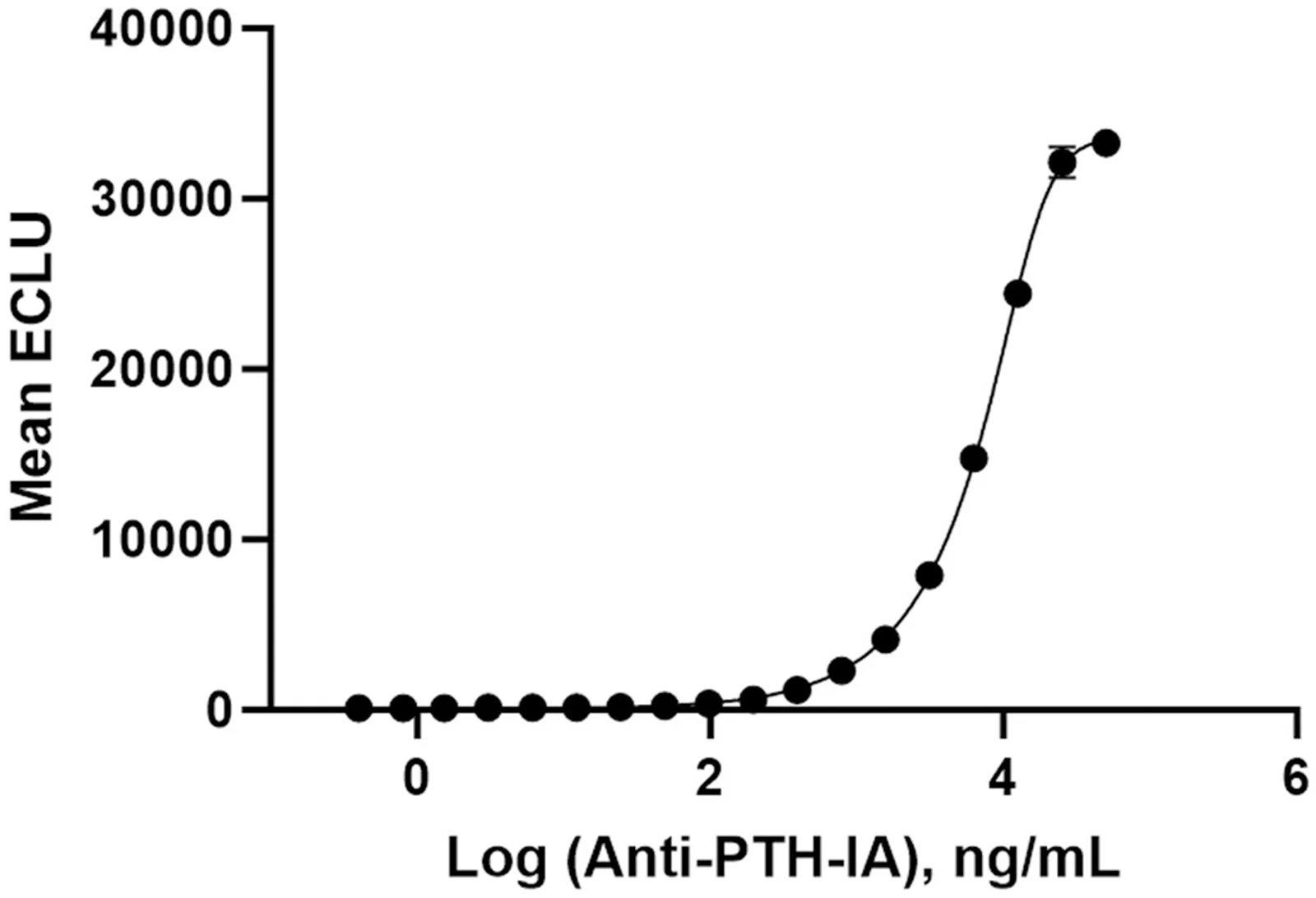
Interpolation of screening assay sensitivity. A pool of 50 drug-naïve human plasma samples spiked with anti-PTH-IA rat polyclonal positive control at different concentrations was assayed in two independent runs and mean results fitted to a 5-PL curve.

The sensitivity of the screening assay was determined based on a rat polyclonal anti-PTH-IA antibody positive control (PC). To construct a sensitivity curve, undiluted pooled human plasma was spiked with 50,000 ng/mL anti-PTH-IA antibody followed by two-fold serial dilutions with pooled human plasma (blank matrix) down to 0.4 ng/mL. The spiked plasma samples were assayed in two independent runs at the MRD of 1:3 and five parameter logistic (5-PL) regression (Figure 3) was used to interpolate the concentration of PTH-IA antibody that corresponds to the plate’s floating SCP. The floating SCP was calculated by multiplying the NC of the plate by a CPF of 1.055 determined during the evaluation of the screening assay cut point on a total of 50 human plasma samples. The interpolated sensitivity was determined to be 3.61 ng/mL, well below the FDA-recommended sensitivity of 100 ng/mL [20], demonstrating that the assay is sufficiently sensitive to detect low-level ADA positive samples.

### 3.5. Confirmatory assay cut point

**Figure 4.**
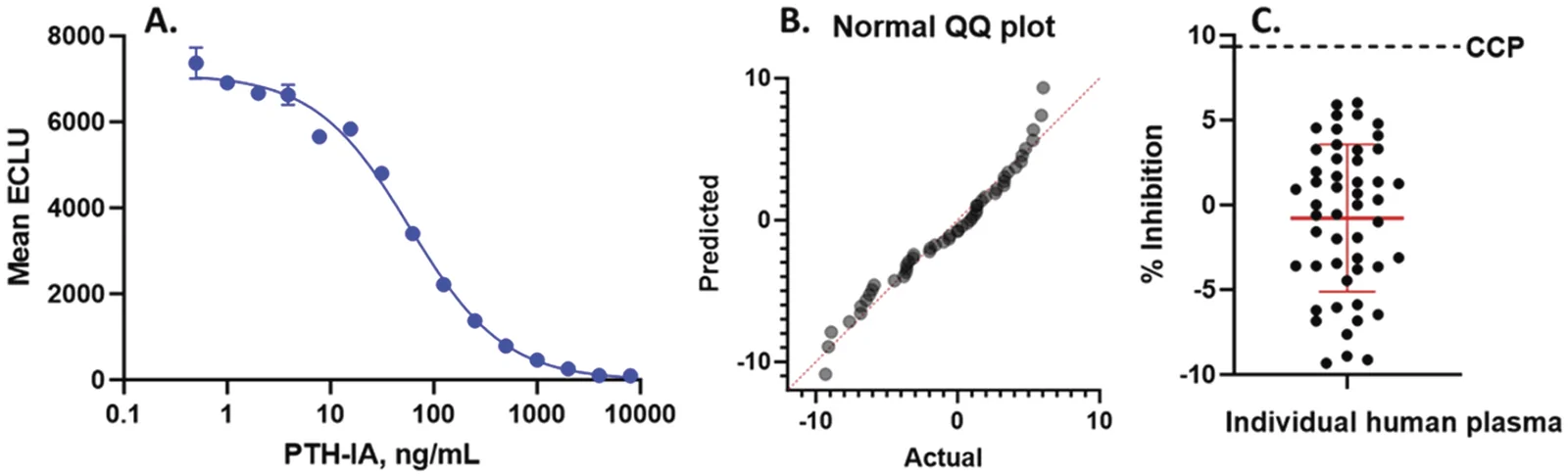
Confirmatory assay cut point calculation. A pool of drug-naïve human plasma samples spiked with rat anti-PTH-IA antibody at 1250 ng/mL was incubated with increasing concentration of unlabeled PTH-IA to determine the concentration of PTH-IA that inhibits signal to levels below SCP (A). Human plasma samples from 50 healthy drug-naïve human donors assayed in the presence and absence of 4 μg/mL PTH-IA (B, C), QQ plot of actual distribution of inhibitory ratios versus predicted normal distribution (B), Scatter plot of percent inhibition with mean ± SD in red. Dashed line indicates confirmatory assay cut point (CCP).

The assay’s ability to detect antibodies specific to PTH-IA was evaluated using a competitive inhibition approach [19] in which signal values from drug-naïve human plasma samples preincubated with label-free PTH-IA were compared with those from unspiked samples. To establish the appropriate concentration of PTH-IA for the competition assay, a pool of 50 drug-naïve human plasma samples was spiked with PC at 1250 ng/mL. The spiked human plasma pool was incubated with two-fold dilutions of 8000 ng/mL unlabeled PTH-IA down to 2 ng/mL. The ECL values from the PC were below the screening assay cut point when preincubated with 4000 ng/mL PTH-IA or above (Figure 4(A)). To determine the confirmatory cut point (CCP), 50 individual human plasma samples similar to those used in the SCP experiment were assayed in duplicate in two independent runs in the presence or absence of the predetermined 4000 ng/mL PTH-IA. The percentage signal inhibition by PTH-IA was determined using the equation by Shankar et al. [19]:

%signal inhibition=100*[1−(average PTH−IA spiked sample/average unspiked sample)]

The ROUT analysis identified no outliers in percentage signal inhibition, and the data exhibited a normal distribution as confirmed by the QQ plot (Figure 4(B)) and the Shapiro–Wilk normality test (*p* = 0.0652). The CCP was determined to be 9.34% using a parametric approach at 1% false-positive error rate (mean +2.33 * SD).

### 3.6. Precision of the screening and confirmatory assays

To evaluate reproducibility of the screening assay, we analyzed NC and three levels of positive control assayed in six wells. The PC was spiked in a pool of drug-naïve plasma samples at 1250 ng/mL for HPC, 100 ng/mL for MPC, and 12.5 ng/mL for LPC. As shown in Table 1, CV for all controls varied between 2.3 % and 5.6% for intra-assay and 3.6 to 16.4% for inter-assay, establishing reproducibility of the screening assay.

We also evaluated the reproducibility of the confirmatory assay by employing HPC at 1250 ng/mL, MPC at 100 ng/mL and LPC at 12.5 ng/mL in the presence or absence of 4 μg/mL PTH-IA. All PTH-IA – spiked PC samples produced signals that showed inhibition above the determined CCP of 9.34%, which confirmed the expectation that ADA positive sample inhibition signal should exceed drug-inhibited response in drug-naïve population [30]. CV% for all controls was within acceptable range as they were below 20% (Table 2) and demonstrated repeatability of the confirmatory assay.

### 3.7. Drug tolerance and effects of other potential assay interferents

PTH-IA present in patient samples can interfere with anti-drug antibody detection due to ADA binding competition between drug and Bt-PTH-IA/St-PTH-IA assay reagents. This would lead to false negative ADA results [28]. We therefore assessed the level of free PTH-IA in plasma at which the 10 ng/mL PC exhibited a positive signal. We spiked PC at 10 ng/mL in a pool of drug-naïve human plasma in the presence of PTH-IA concentrations ranging from 1 μg/mL to 0.5 ng/mL (Table 3). The floating cut point was determined to be 113.3 ECLU and drug tolerance was interpolated from a 5-PL nonlinear curve and determined to be 27.3 ng/mL.

We also considered other potential interferents that could affect ADA assessment. PTH-IA is a derivative of PTHrP which shares amino acid sequences with PTH [12,39]. Both PTHrP and PTH are soluble endogenous peptides that, if sufficiently present in patient samples, can cross-react with PTH-IA antibodies thereby leading to false negative ADA assay results. To evaluate potential interference of these PTH-IA related peptides, we compared ECL assay signals generated by a pool of drug-naïve human plasma supplemented with 10 ng/mL anti-PTH-IA positive control antibody in the absence or presence of 1 ng/mL of PTH or PTHrP alone or in combination. The normal levels of endogenous PTH and PTHrP in circulation are at least 50-fold lower than the 1 ng/mL used in the assay [40]. The acceptance criterion for noninterference was set at ±20% of the ECL signal obtained from PC alone. As shown in Table 4, all samples with PC tested positive and neither PTH nor PTHrP as single agent or in combination interfered with the detection of PTH-IA positive control antibody.

## 4. Discussion

Immunogenicity assessment is critical for the accurate interpretation of efficacy and safety data in preclinical and clinical studies of biotherapeutics. It also supports the development of mitigation strategies to manage treatment-induced immune responses. The objective of this study was to develop a bridging anti-drug antibody assay using an electrochemiluminescence-based platform to support the immunogenicity evaluation of a PTH inverse agonist peptide (PTH-IA) under development for the treatment of JMC, an ultra-rare genetic disease of skeletal development and mineral ion homeostasis.

To demonstrate ADA-drug product binding, we used PTH-IA itself in the generation of biotinylated and ruthenylated critical reagents. We encountered two challenges in the conjugation of PTH-IA using EZ-Link Sulfo-NHS-Biotin to produce a capture reagent and MSD GOLD SULFO-TAG NHS-Ester to generate a detection reagent. 1X PBS at pH 7–9, the routinely used phosphate-buffered saline for biotinylation and ruthenylation, did not dissolve PTH-IA. We overcame this solubility issue by performing the conjugation in 30% DMSO in 1X PBS at pH 8. Although this mixture completely dissolved PTH-IA and free EZ-Link Sulfo- NHS-biotin, biotinylated PTH-IA precipitated out of solution. Analysis of the precipitate by LC-MS confirmed an added mass of 452 Da to PTH-IA (Figure 1(F)) which is consistent with incorporation of two biotin labels. This small difference in molecular weights of free and labeled PTH-IA was the second challenge as it made separation of Bt-PTH-IA from free PTH-IA by size exclusion chromatography impractical. We exploited the differential solubility of free PTH-IA and Bt-PTH-IA in 30% DMSO in 1X PBS and isolated the capture reagent at a high purity of 99.8%. Residual unlabeled PTH-IA was 0.2%. This level was 135-fold lower than the 27.3 ng/mL drug tolerance and was deemed too low to interfere with the assay performance. Residual biotin can compete with Bt-PTH-IA for the binding of streptavidin coated plate thereby reducing the assay signal. Analysis of LC-MS data established absence of a chromatographic peak of residual unreacted biotin in the Bt-PTH-IA pellet sample.

In contrast to neutral biotin moieties, which eliminated the positive charges on lysine residue and amino terminus of PTH-IA and thereby reducing solubility, conjugation with negatively charged MSD GOLD SULFO-TAG NHS-Ester did not result in precipitation. A challenge ratio of 2:1 resulted in minimum formation of doubly ruthenylated PTH-IA, with unmodified PTH-IA remaining the predominant species in the heterogeneous mixture. Free PTH-IA was unwanted as it could compete with conjugates for ADA binding and adversely impact assay performance. Increasing the conjugation ratio to 10:1 drove the reaction to completion, resulting in maximum incorporation of two sulfo-tags per PTH-IA molecule. The high sulfo-tag incorporation was desirable as it was expected to result in higher ECL signals.

The current optimized assay met acceptable performance criteria such as precision and sensitivity based on the anti-PTH-IA positive control. However, it lacks hook effects assessment, and this is a limitation of this study. Although no evidence of signal reduction was observed at the highest anti-PTH-IA concentration (50,000 ng/mL) tested in the calibration curve, future investigations should evaluate further hook effect as well as dilutional linearity.

## 5. Conclusion

The capture and detection reagents generated through optimized amine coupling conjugation processes were critical to the development of an ADA assay with adequate sensitivity, low background, and robust positive control performance. These reagents also enabled the establishment of decision-enabling screening and confirmatory cut point parameters (CPF and CCP) and demonstrated the suitability of the assay for the detection of anti-PTH-IA antibodies in human plasma.

## Figures and Tables

**Table 1.** Precision of screening assay (*n* = 6).

QC level	NC	LPC	MPC	HPC
Concentration of PC (ng/mL)	0	12.5	100	1250
		**Intra-assay precision**		
Mean (ECLU)	109.5	168.3	567.3	5191.8
SD	6.2	3.9	15.6	124.2
%CV	5.6	2.3	2.7	2.4
		**Inter-assay precision**		
Mean (ECLU)	105.8	150.8	623.5	5326.3
SD	5.3	24.7	79.4	190.1
%CV	5.0	16.4	12.7	3.6

**Table 2.** Precision of confirmatory assay (*n* = 6).

QC level	LPC	MPC	HPC
Concentration of PC (ng/mL)	12.5	100	1250
		**Intra-assay precision**	
Mean (% Inhibition)	31.5	80	97.4
SD	4.1	0.8	0.1
%CV	13	0.9	0.1
		**Inter-assay precision**	
Mean (% Inhibition)	32.0	81.2	97.3
SD	0.5	1.7	0.2
%CV	1.7	2.1	0.2

**Table 3.** Determination of drug tolerance.

Drug concentration (ng/mL)	PC (ng/mL)	Mean ECLU	Result
1000.0	10	104.5	Negative
500.0	10	107.8	Negative
250.0	10	106.8	Negative
125.0	10	109.3	Negative
62.5	10	109.2	Negative
31.3	10	113.2	Negative
15.6	10	114.7	Positive
7.8	10	119.8	Positive
3.9	10	120.8	Positive
2.0	10	124.5	Positive
1.0	10	125.0	Positive
0.5	10	129.5	Positive
0	10	140.5	Positive

**Table 4.** Evaluation of assay interference by PTH and PTHrP.

Interferent	Mean ECLU at 10 ng/mL PC	% recovery
1 ng/mL PTH	144.0	107.1
1 ng/mL PTHrP	133.7	99.4
Combination of PTH and PTHrP at 1 ng/mL each	130.2	96.8
No interferent	134.5	100

## Data Availability

The data supporting the findings of this study are available within the article.

## Article highlights

- PTH-IA is a novel synthetic peptide that is being evaluated as a treatment for Jansen’s metaphyseal chondrodysplasia.
- We developed an electrochemiluminescence bridging assay to detect antibodies directed against PTH-IA in human plasma.
- Capture and detection reagents were generated by amine-coupling chemistry and characterized by HPLC-TOF mass spectrometry.
- The assay demonstrated high sensitivity (low detection limit was 3.61 ng/mL) and excellent precision (intra-assay and inter-assay CV: 2.3–16.4%).
- The confirmatory cut point was 9.34% inhibition, drug tolerance was 27.3 ng/mL, and the assay exhibited a high specificity: parathyroid hormone (PTH) and parathyroid hormone-related peptide (PTHrP), which are structurally similar to PTH-IA, did not interfere with the performance of the assay.
